# Exploring the Relationship Between Nursing Staff and Family Members’ Appraisal of Resident Care in Nursing Homes: The Role of Facility Ownership

**DOI:** 10.3390/nursrep15020064

**Published:** 2025-02-11

**Authors:** Roberto J. Millar, Christin Diehl, Nancy Kusmaul, Ian Stockwell

**Affiliations:** 1 The Hilltop Institute, University of Maryland Baltimore County, 1000 Hilltop Cir., Baltimore, MD 21250, USA; cdiehl@hilltop.umbc.edu; 2 School of Social Work, University of Maryland Baltimore County, 1000 Hilltop Cir., Baltimore, MD 21250, USA; nkusmaul@umbc.edu; 3 Department of Information Systems, University of Maryland Baltimore County, 1000 Hilltop Cir., Baltimore, MD 21250, USA; istock1@umbc.edu

**Keywords:** long-term care, nursing home, family engagement, nurse qualification

## Abstract

**Background/Objectives:** To address long-standing staffing challenges and elevating care standards in the United States, new legislation will require a minimum of 0.55 h per resident day (HPRD) of registered nurse (RN) care, 2.45 HPRD of certified nursing aide (CNA) care, and a combined total of 3.48 HPRD across any combination of nursing staff. We examine differences in family members’ views of care quality between facilities meeting the minimum staffing requirements and those that do not and whether there is any difference in those associations by facility ownership. **Methods:** This cross-sectional study utilized public data from 218 Medicare and Medicaid-certified nursing facilities in Maryland, collected in 2023. We used regression analyses to examine the association between staffing requirements and quality of care ratings, considering facility ownership status as a potential moderator. **Results:** Compared to facilities with CNA staffing levels below the cut off, facilities that met the CNA staffing requirement were rated more favorably by family members in overall quality and across the subdomains of staffing, care, activities, and security. In contrast, meeting the RN 0.55 cut off was not associated with family ratings across any quality domain. A facility for-profit status did not moderate the relationship between staffing and family ratings. **Conclusions:** These results suggest that CNA staff time is a significant driver of care quality and that non-profit facilities may already be closer to meeting new federal requirements. These findings highlight the need for regulations that support the minimum nursing staffing requirements to enhance care quality. Future research should identify the specific factors contributing to higher quality care in non-profit facilities and explore ways to implement these practices in for-profit settings.

## 1. Introduction

Nursing homes, or nursing facilities, provide crucial services and support to individuals who need around-the-clock assistance due to chronic illness or disability. The services available to nursing facility residents include medical care, rehabilitation, and assistance with activities of daily living (ADLs), such as dressing and bathing, all aimed at ensuring the health, safety, and well-being of residents [1,2]. Central to effective care in these facilities is the concept of person-centered care, which prioritizes the individual needs and preferences of each resident [3]. This approach involves creating personalized care plans, fostering meaningful connections between caregivers and residents, and importantly, engaging family members in the care process. For instance, family involvement may promote residents’ well-being, allow for continuity of care, and facilitate communication between residents and the caregiving team [3,4].

Registered nurses (RNs), licensed practical nurses (LPNs), and certified nursing aides (CNAs) comprise the primary care team in nursing facilities, and each hold distinct responsibilities in the care of residents [1,5]. RNs oversee clinical care, develop care plans, administer medications, and perform complex medical procedures while supervising other nursing staff, while LPNs support RNs by administering medications, conducting wound care, and monitoring health, often assuming supervisory duties to ensure CNAs effectively contribute to resident care plans. CNAs provide most of the direct care to residents, including assistance with ADLs, monitoring vital signs, and notifying the RN or LPN of any changes in the resident’s condition [5]. The composition of the nursing staff care team has been shown to contribute to resident outcomes and well-being. In general, a higher nursing staff capacity and skill mix are linked to better quality of care and quality of life of residents [6,7,8].

In the United States (U.S.), federal and state authorities regulate nurse staffing requirements in nursing facilities, including staff qualifications and staffing capacity. The 1987 Nursing Home Reform Act, overseen by the Centers for Medicare & Medicaid Services (CMS), mandates that facilities maintain sufficient nursing staff to meet residents’ needs, with states empowered to impose additional regulations [5]. In April 2024, the CMS released new regulations aimed at addressing long-standing staffing challenges and elevating care standards for the nearly 1.2 million nursing facility residents across the country [9]. These reforms included stricter staffing ratios and minimum hours of care, as well as increased transparency regarding staffing levels. Per the final rule, all facilities must meet the following requirements by 2029: a minimum of 0.55 h per resident day (HPRD) of RN care, 2.45 HPRD of NA care, and a combined total of 3.48 HPRD across any combination of nursing staff (i.e., RN, LPN, and CNA) [9]. To mitigate workforce challenges, facilities in rural areas are granted additional time to come into compliance. Taken together, these efforts are to ensure nursing facilities have reasonably adequate personnel to deliver high-quality care and ultimately improve the quality of care provided to residents.

Preliminary reporting indicates that fewer than one in five facilities in the U.S. would meet the staffing requirements as of April 2024 [10]. Moreover, there are stark differences in the proportion of for-profit and non-profit facilities that would meet the requirements; approximately 11% of for-profit facilities and 41% of non-profit facilities would meet the daily nursing hours staffing requirements [10]. These findings align with research noting poorer quality of care in for-profit nursing facilities compared to non-profit facilities [8]. Compared to for-profit facilities, non-profit facilities generally perform better in regulatory assessments and are rated higher by consumers [11]. Several contextual factors have been linked to the higher care quality generally observed in non-profit over for-profit facilities. For instance, non-profit facilities generally pay higher wages to nursing staff and hire a greater number of staff per resident [12,13]. Higher wages and less strain on the nursing staff may contribute to lower staff turnover and greater job satisfaction, which, in turn, can contribute to better care for its residents.

In this study, we examine the association between a facility meeting the minimum nursing staffing requirements and family members’ views of the quality of care provided to its residents. Furthermore, we explore these associations in relation to the facility for-profit status. We test the following hypotheses: (a) Facilities that meet one or both staffing requirements receive higher quality ratings from family members than those that do not. (b) Any associations between meeting staffing requirements and family ratings are stronger for for-profit facilities compared to non-profit facilities.

## 2. Materials and Methods

### 2.1. Study Design and Sample

This cross-sectional research study draws from two publicly available, facility-level data sources of all 218 facilities (of 225) certified to serve Medicare and Medicaid beneficiaries in the state of Maryland in 2023 and for which data were collected.

### 2.2. Data Sources and Measures

#### 2.2.1. Dependent Variables

The Nursing Home Family Experience of Care Survey, developed by the Maryland Health Care Commission (MHCC), is intended to evaluate perceptions of care quality from family members, care partners, and other authorized representatives of nursing facility residents with stays of 100 days or greater [14]. Administered annually, a convenience sample of respondents are asked to reflect on the last six months of care and provide an overall quality rating (i.e., overall satisfaction with care and responses to items encompassing four domain-specific measures (i.e., staff and administration, care provided, activities, and security/personal rights). The MHCC aggregates survey items to produce valid and reliable measures of family views of care across distinct quality domains. The ratings for the overall measure range from 1 to 10, while the domain-specific ratings range from 1 to 4, with higher ratings representing more positive views of care. In this 2023 cycle, there were nearly 26 respondents per facility, with an average response rate of 27% across all 218 facilities. For the current research study, we focused on family members’ overall rating of the facility, as well as ratings for the staff, care, activities, and security subdomains.

#### 2.2.2. Independent Variables and Covariates

We used CMS’ Five Star Nursing Home Quality Rating System ProviderInfo files to determine the average nurse staffing hours of care by facility, facility ownership, nurse staff turnover, facility size, and geographic location. These archived monthly data files contain data on all Medicare and Medicaid-certified nursing facilities in the U.S., including acuity-adjusted HPRD for RNs, CNAs, and LPNs [15]. Acuity-adjusted HPRD accounts for a resident’s intensity of care needs and can assist nursing facilities with determining the adequate staffing levels to support the residents’ individualized needs and well-being. RN HPRD and CNA HPRD were dichotomized based on the CMS guidelines to indicate whether the facility met the cut offs of 0.55 RN HPRD or 2.45 NA HPRD. Additionally, we created a covariate indicator of whether facilities met an additional 0.48 of LPN HPRD to align with the total 3.48 HPRD requirement. Ownership type was classified as for-profit or non-profit. The overall nursing staff turnover was determined as the percentage of nursing staff that left the facility within the last 12 months; facilities with 50% or greater turnover were classified as having high nursing staff turnover. We categorized facility size based on the average number of residents per day (i.e., small (1–60), medium (61–120), and large (121+)). Finally, facilities were classified as urban/rural based on Maryland’s Nursing Facility Services Regional Alignment, a standard designation for the state’s facilities.

### 2.3. Statistical Analyses

Using national provider identifiers, we merged Family Experience of Care Survey responses and CMS’ ProviderInfo files to construct a facility-level data set of nursing facilities operating in the state in December 2023. To test our first hypothesis, we used a series of unadjusted and adjusted ordinary least square (OLS) regressions to examine the association between meeting the proposed nursing staffing cut offs and family members’ quality ratings. OLS regression was selected as the analytic method given the continuous nature of the independent variables and based on preliminary analyses indicating that the assumptions of linearity, independence, homoscedasticity, and normality were met. To test our second hypothesis, we explored models with interaction terms to examine the potentially moderating effect of ownership status on these relationships. All analyses were conducted using SAS version 9.4 (Copyright © 2013, SAS Institute Inc., Cary, NC, USA). An alpha threshold of *p* < 0.05 was used for hypothesis testing.

## 3. Results

### 3.1. Descriptive

On a scale from 1 to 10, with 10 indicating greater satisfaction with care, the average family rating for all the facilities was 7.44 (SD = 1.12) (Table 1). The for-profit facilities had a lower average rating of 7.16 (SD = 0.99), while the non-profit facilities had a higher average rating of 8.25 (SD = 1.07). Similarly, family members rated non-profit facilities higher than for-profit facilities across the staff, care, activities, and security subdomains—each ranging from 1 to 4, with higher scores indicating greater satisfaction. Over 60% of the facilities had RN HPRD of 0.55 or higher, while nearly 14% of facilities met the CNA cut off of 2.45 HPRD. Non-profit facilities were more likely than for-profit facilities to meet both cut offs for the RN and CNA requirements. Finally, a greater proportion of for-profit facilities were large, while there were no differences observed in the distribution of urban facilities across ownership type.

### 3.2. Inferential

Table 2 shows the unadjusted (β) and adjusted (adj. β) OLS regression coefficients of the association between meeting the minimum nurse staffing requirements, ownership status, and family quality of care ratings across five quality domains. Across all five unadjusted models, meeting the NA HPRD cut off was positively associated with higher quality ratings, while a for-profit status was linked to lower quality ratings. These associations remained, albeit to a lesser extent, as indicated by the coefficients, in models adjusted for covariates. Meeting the RN HPRD was only statistically significantly associated with the overall quality ratings in the unadjusted model (β = 0.30, standard error (SE) = 0.13; *p* < 0.05); this association was no longer significant in the models adjusted for covariates (adj. β = 0.21, SE = 0.12; *p* ≥ 0.05).

Table 3 shows adjusted regression coefficients of the association between meeting the minimum nurse staffing requirements and family quality of care ratings, accounting for the interaction of each with ownership status. The introduction of interaction terms between nurse staffing requirement adherence and facility ownership status showed a non-significant moderation of the associations observed in previous models.

## 4. Discussion

The nursing staff comprises the primary care team with whom residents and family members interact in nursing facilities, and research indicates that higher nursing staff capacity and skill mix (e.g., diverse certification and training backgrounds) are linked to better quality of resident care [7,8]. In efforts to address long-standing staffing challenges and elevate care standards in facilities across the country, the U.S. CMS introduced new legislation in April 2024 that requires a minimum of 0.55 HPRD of RN care, 2.45 HPRD of NA care, and a combined total of 3.48 HPRD across any combination of nursing staff [9]. We used publicly available data on nursing facilities in the state of Maryland to determine whether facilities meeting the proposed minimum RN and CNA HPRD standards (i.e., 0.55 RN HPRD and 2.45 CNA HPRD) receive higher quality ratings by residents’ family members. We then examined these associations in the context of facility ownership status (i.e., for-profit and non-profit).

We hypothesized that facilities that meet one or both staffing requirements receive higher quality ratings from family members than those that do not. The results showed that, compared to facilities with CNA staffing levels below the 2.45 HPRD cut off, facilities that met the CNA staffing requirement were rated more favorably by family members in overall quality, as well as across the subdomains of staffing, care, activities, and security. In contrast, meeting the RN 0.55 cut off was not associated with family ratings across any quality domain. This trend likely reflects the hands-on nature of CNAs’ care responsibilities. CNAs provide most of the direct care to residents and are more likely to interact with residents and their family members than RNs [5]. The visibility of CNAs in the care environment and their greater involvement with daily care relative to RNs may therefore be viewed by residents and their families more favorably.

Our second hypothesis was that any associations between meeting staffing requirements and family ratings are stronger for for-profit facilities compared to non-profit facilities. While non-profit facilities were more likely to meet both staffing cut offs and be rated more favorably by family members, we found no evidence that ownership status moderated the relationship between meeting the staffing requirements and family members’ ratings. Contextual factors like higher pay for nursing staff have been linked to lower staff turnover rates and overall better outcomes seen in residents of non-profit facilities [12,13]. Since non-profit facilities are more likely to meet the staffing requirements and generally provide higher quality care, policies could be designed to support and expand non-profit nursing facilities. This could include financial incentives or grants to encourage the establishment and operation of non-profit facilities.

Our findings align with the existing literature that highlights the disparity in care quality between for-profit and non-profit nursing facilities. Previous studies have consistently shown that non-profit facilities tend to provide higher quality care compared to their for-profit counterparts. For instance, research has indicated that non-profit facilities are more likely to meet staffing requirements and have lower staff turnover rates, which are critical factors in delivering high-quality care [8,10]. Our results corroborate earlier findings and suggest that the ownership status of a facility plays a crucial role in determining the quality of care provided to residents. Given the significant differences in care quality between for-profit and non-profit nursing facilities, it is imperative for policymakers to consider these findings when developing regulations and standards for nursing homes. Future research should focus on identifying the specific factors that contribute to higher quality care in non-profit facilities and exploring ways to implement these practices in for-profit settings. Additionally, stakeholders should advocate for policies that support adequate staffing levels and reduce turnover rates to ensure that all residents receive the highest standard of care.

There are several limitations worth considering when interpreting these findings. First, the cross-sectional design of this research limits assumptions of causality between staffing level and quality ratings. Longitudinal research designs may be used to observe staffing trends and their influence on care quality over time, providing more concrete evidence of causal relationships between the two. Second, the data are limited to facilities in the state of Maryland, which may limit the generalizability of our findings to a wider population. Maryland is one of three states (the others being Ohio and Minnesota) that systematically collect data on family and/or resident satisfaction annually. Each state uses a different sampling and data collection method, which limits data aggregation for a larger, more geographically diverse sample. Nevertheless, we believe that the sociodemographic diversity in the region and average response rate of 26 per facility compensates for this limitation. A final limitation worth noting is the exclusion of residents’ own views of care. While family views can provide valuable insight into the quality of care in the facility, the inclusion of residents’ views, preferences, and perspectives should be incorporated whenever possible [1,3].

## 5. Conclusions

The implications of these findings are critical for policymakers and stakeholders in the nursing home industry, as they underscore the need for regulations that support adequate staffing and reduce turnover rates to improve care quality. It is essential that, as the new minimum staffing requirements become effective, there are additional efforts in Maryland and beyond to systematically assess how staffing trends influence care quality outcomes. Moreover, future research should continue to explore the specific factors that contribute to higher quality care in non-profit facilities and investigate ways to implement these practices in for-profit settings.

## Figures and Tables

**Table 1:** Descriptive statistics for all the facilities and by ownership type.

	Total (N = 218)		Ownership Type				Range
			For-Profit (n = 162)		Non-Profit (n = 56)		
Family Ratings, M (SD)							
Overall	7.44	(1.12)	7.16	(0.99)	8.25	(1.07)	(1–10)
Staff	3.31	(0.27)	3.25	(0.25)	3.49	(0.27)	(1–4)
Care	3.26	(0.32)	3.19	(0.30)	3.48	(0.26)	(1–4)
Activities	2.84	(0.42)	2.77	(0.38)	3.07	(0.43)	(1–4)
Security	3.24	(0.33)	3.16	(0.30)	3.47	(0.29)	(1–4)
Nursing Staff Requirement, n (%)							
≥0.55 RN HPRD	133	(61.01%)	84	(51.85%)	49	(87.50%)	(0–1)
≥2.45 CNA HPRD	30	(13.76%)	12	(7.41%)	18	(32.14%)	(0–1)
Covariates, n (%)							
≥0.48 LPN HPRD	204	(93.58%)	152	(93.83%)	52	(92.86%)	(0–1)
High Nursing Staff Turnover	96	(44.04%)	81	(50.00%)	15	(26.79%)	(0–1)
Small Size	27	(12.39%)	12	(7.41%)	15	(26.79%)	(0–1)
Medium Size	84	(38.53%)	62	(38.27%)	22	(39.29%)	(0–1)
Large Size	107	(49.08%)	88	(54.32%)	19	(33.93)	(0–1)
Urban	183	(83.94%)	136	(83.95%)	47	(83.93%)	(0–1)

Note: M = mean; SD = standard deviation; RN = registered nurse; CNA = certified nursing aide; LPN = licensed practical nurse; HPRD = hour per resident day. A high nursing staff turnover indicates 50% or greater of the nursing staff left the facility within the last 12 months; facility size is based on the average number of residents per day (i.e., small (1–60), medium (61–120), and large (121+)); urban designation is based on Maryland’s Nursing Facility Services Regional Alignment.

**Table 2:** Ordinary least squares regression models of the association between meeting the minimum nurse staffing requirements and family quality of care ratings.

	Overall		Staff		Care		Activities		Security	
	(1–10)		(1–4)		(1–4)		(1–4)		(1–4)	
	β (SE)	adj. β (SE)	β (SE)	adj. β (SE)	β (SE)	adj. β (SE)	β (SE)	adj. β (SE)	β (SE)	adj. β (SE)
Nursing Staff Requirement										
≥0.55 RN HPRD	0.30 (0.13) *	0.21 (0.12)	0.03 (0.04)	0.01 (0.03)	0.04 (0.04)	0.01 (0.03)	0.07 (0.05)	0.03 (0.05)	0.07 (0.04)	0.05 (0.03)
≥2.45 CNA HPRD	0.93 (0.20) ***	0.63 (0.13) **	0.21 (0.06) ***	0.14 (0.05) **	0.25 (0.06) ***	0.15 (0.05) **	0.31 (0.08) ***	0.17 (0.07) *	0.21 (0.06) **	0.12 (0.05) *
										
Ownership Type										
Non-Profit (*ref.*)										
For-Profit	−0.79 (0.15) ***	−0.51 (0.14) **	−0.18 (0.04) ***	−0.11 (0.04) **	−0.22 (0.04) ***	−0.15 (0.04) **	−0.21 (0.06) ***	−0.15 (0.05) *	−0.24 (0.04) ***	−0.16 (0.04) ***
										
Covariates										
≥0.48 LPN HPRD		0.17 (0.22)		0.04 (0.06)		0.12 (0.06)		0.01 (0.09)		−0.01 (0.06)
High Nursing Turnover		−0.61 (0.12) ***		−0.15 (0.06) ***		−0.17 (0.03) ***		−0.05 (0.04)		−0.15 (0.03) **
Large Size (*ref.*)										
Small Size		1.35 (0.20) ***		0.33 * (0.06) **		0.44 (0.05) ***		0.60 (0.08) ***		0.42 (0.06) ***
Medium Size		0.45 (0.12) ***		0.08 (0.03) *		0.14 (0.03) ***		0.17 (0.05) **		0.11 (0.03) **
Urban		−0.51 (0.15) **		−0.16 (0.04) ***		−0.16 (0.04) ***		−0.17 (0.06) **		−0.19 (0.04) ***
Adjusted R^2^	0.28	0.45	0.21	0.39	0.23	0.45	0.17	0.36	0.23	0.42
Model F	27.35	21.46	19.17	17.04	21.83	21.32	14.29	14.47	21.87	19.3

Note: β = unadjusted beta coefficient; adj. β = covariate-adjusted beta coefficient; SE = standard error; ref = reference group; RN = registered nurse; CNA = certified nursing aide; LPN = licensed practical nurse; HPRD = hour per resident day. A high nursing staff turnover indicates 50% or greater of the nursing staff left the facility within the last 12 months; facility size is based on the average number of residents per day (i.e., small (1–60), medium (61–120), and large (121+)); urban designation is based on Maryland’s Nursing Facility Services Regional Alignment; * *p* < 0.05, ** *p* < 0.01, and *** *p* < 0.001.

**Table 3:** Adjusted ordinary least square regression models predicting ratings from family members by ownership type.

	Overall	Staff	Care	Activities	Security
	adj. β (SE)	adj. β (SE)	adj. β (SE)	adj. β (SE)	adj. β (SE)
Nursing Staff Requirement					
≥0.55 RN HPRD	0.15 (0.12)	0.02 (0.03)	−0.06 (0.06)	0.06 (0.05)	0.01 (0.03)
≥2.45 NA HPRD	0.53 (0.03) *	0.11 (0.02) *	0.10 (0.08)	0.17 (0.10)	0.07 (0.05)
					
Ownership Type					
Non-Profit (*ref.*)					
For-Profit	−0.6 (0.06)	−0.11 (0.13)	−0.24 (0.06) *	−0.13 (0.11)	−0.22 (0.10) *
					
Covariates					
≥0.55 LPN HPRD	−0.01 (0.03)	0.01 (0.01)	0.13 (0.10)	−0.1 (0.02)	−0.01 (0.01)
High Nursing Staff Turnover	−0.01 (0.01) **	−0.04 (0.02) ***	−0.01 (0.01) **	0.05 (0.02)	−0.03 (0.01) **
Small Size	1.24 (0.10) ***	0.29 (0.12) ***	0.42 (0.14) ***	0.59 (0.07) ***	0.39 (0.05) ***
Medium Size	0.31 (0.02) *	0.05 (0.03)	0.10 (0.04) *	0.13 (0.02) *	0.08 (0.06)
Urban	−0.63 (0.03) **	−0.18 (0.02) **	−0.19 (0.07) **	−0.15 (0.08) *	−0.22 (0.02) ***
					
Interactions					
≥0.55 RN HPRD * For-Profit	0.13 (0.14)	−0.01 (0.03)	0.09 (0.08)	−0.01 (0.01)	0.07 (0.04)
≥2.45 NA HPRD * For-Profit	0.29 (0.07)	0.13 (0.07)	0.13 (0.03)	−0.03 (0.01)	0.13 (0.10)
Adjusted R^2^	0.43	0.41	0.43	0.33	0.41
Model F	13.49	11.96	13.16	8.68	12.28

Note: RN = registered nurse; CNA = certified nursing aide; LPN = licensed practical nurse; HPRD = hour per resident day. A high nursing staff turnover indicates 50% or greater of the nursing staff left the facility within the last 12 months; facility size is based on the average number of residents per day (i.e., low (1–60), medium (61–120), and high (121+)); urban designation is based on Maryland’s Nursing Facility Services Regional Alignment; * *p* < 0.05, ** *p* < 0.10, and *** *p* < 0.001.

## Data Availability

The data used in this manuscript come from publicly available sources. The Maryland Family Satisfaction survey can be accessed at https://healthcarequality.mhcc.maryland.gov/NursingHome/List?sCol=name&sDir=ASC (accessed on 6 November 2024). CMS data can be accessed at https://data.cms.gov/provider-data/archived-data/nursing-homes. (accessed on 6 November 2024).

## Public Involvement Statement

Family members of Maryland nursing facilities in Maryland with stays of over 100 days were identified by MHCC and surveyed.

## Guidelines and Standards Statement

The drafting of this manuscript was informed by the STROBE checklist for cross-sectional studies.
